# On Well-Founded and Recursive Coalgebras

**DOI:** 10.1007/978-3-030-45231-5_2

**Published:** 2020-04-17

**Authors:** Jiří Adámek, Stefan Milius, Lawrence S. Moss

**Affiliations:** 8 ENS/CNRS, Université Paris-Saclay, Cachan, France; 9 University of Duisburg-Essen, Duisburg, Germany; 10 Czech Technical University, Prague, Czech Republic; 11 Friedrich-Alexander-Universität Erlangen-Nürnberg, Erlangen, Germany; 12 Indiana University, Bloomington, IN USA

**Keywords:** Well-founded, Recursive, Coalgebra, Initial Algebra, General Recursion Theorem

## Abstract

This paper studies fundamental questions concerning category-theoretic models of induction and recursion. We are concerned with the relationship between well-founded and recursive coalgebras for an endofunctor. For monomorphism preserving endofunctors on complete and well-powered categories every coalgebra has a well-founded part, and we provide a new, shorter proof that this is the coreflection in the category of all well-founded coalgebras. We present a new more general proof of Taylor’s General Recursion Theorem that every well-founded coalgebra is recursive, and we study conditions which imply the converse. In addition, we present a new equivalent characterization of well-foundedness: a coalgebra is well-founded iff it admits a coalgebra-to-algebra morphism to the initial algebra.

### 1 Introduction

What is induction? What is recursion? In areas of theoretical computer science, the most common answers are related to *initial algebras*. Indeed, the dominant trend in abstract data types is initial algebra semantics (see e.g. [19]), and this approach has spread to other semantically-inclined areas of the subject. The approach in broad slogans is that, for an endofunctor $F$ describing the type of algebraic operations of interest, the initial algebra $\mu F$ has the property that for every $F$-algebra $A$, there is a unique homomorphism $\mu F \to A$, and this *is* recursion. Perhaps the primary example is *recursion on* $\mathbb{N}$, *the natural numbers*. Recall that $\mathbb{N}$ is the initial algebra for the set functor $FX = X + 1$. If $A$ is any set, and $a \in A$ and $\alpha\colon A \to A + 1$ are given, then initiality tells us that there is a unique $f\colon \mathbb{N} \to A$ such that for all $n \in \mathbb{N}$,

$$f(0) = a \qquad f(n+1) = \alpha(f(n)). \tag{1.1}$$

* A full version of this paper including full proof details is available on arXiv [5].

** Supported by the Grant Agency of the Czech Republic under grant 19-00902S.
*** Supported by Deutsche Forschungsgemeinschaft (DFG) under project MI 717/5-2.
† Supported by grant #586136 from the Simons Foundation.

J. Goubault-Larrecq and B. König (Eds.): FOSSACS 2020, LNCS 12077, pp. 17–36, 2020.
https://doi.org/10.1007/978-3-030-45231-5_2

Then the first additional problem coming with this approach is that of how to "recognize" initial algebras: Given an algebra, how do we really know if it is initial? The answer – again in slogans – is that initial algebras are the ones with "no junk and no confusion."

Although initiality captures some important aspects of recursion, it cannot be a fully satisfactory approach. One big missing piece concerns recursive definitions based on well-founded relations. For example, the whole study of termination of rewriting systems depends on well-orders, the primary example of *recursion on a well-founded order*. Let $(X, R)$ be a well-founded relation, i.e. one with no infinite sequences $\cdots x_2 \, R \, x_1 \, R \, x_0$. Let $A$ be any set, and let $\alpha\colon \mathscr{P}A \to A$. (Here and below, $\mathscr{P}$ is the power set functor, taking a set to the set of its subsets.) Then there is a unique $f\colon X \to A$ such that for all $x \in X$,

$$f(x) = \alpha(\{f(y) : y \; R \; x\}). \tag{1.2}$$

The main goal of this paper is the study of concepts that allow one to extend the algebraic spirit behind initiality in (1.1) to the setting of recursion arising from well-foundedness as we find it in (1.2). The corresponding concepts are those of well-founded and recursive coalgebras for an endofunctor, which first appear in work by Osius [22] and Taylor [23, 24], respectively. In his work on categorical set theory, Osius [22] first studied the notions of well-founded and recursive coalgebras (for the power-set functor on sets and, more generally, the power-object functor on an elementary topos). He defined recursive coalgebras as those coalgebras $\alpha\colon A \to \mathscr{P}A$ which have a unique coalgebra-to-algebra homomorphism into every algebra (see Definition 3.2).

Taylor [23, 24] took Osius' ideas much further. He introduced well-founded coalgebras for a general endofunctor, capturing the notion of a well-founded relation categorically, and considered recursive coalgebras under the name 'coalgebras obeying the recursion scheme'. He then proved the General Recursion Theorem that all well-founded coalgebras are recursive, for every endofunctor on sets (and on more general categories) preserving inverse images. Recursive coalgebras were also investigated by Eppendahl [12], who called them algebra-initial coalgebras. Capretta, Uustalu, and Vene [10] further studied recursive coalgebras, and they showed how to construct new ones from given ones by using comonads. They also explained nicely how recursive coalgebras allow for the semantic treatment of (functional) divide-and-conquer programs. More recently, Jeannin et al. [15] proved the General Recursion Theorem for polynomial functors on the category of many-sorted sets; they also provide many interesting examples of recursive coalgebras arising in programming.

Our contributions in this paper are as follows. We start by recalling some preliminaries in Section 2 and the definition of (parametrically) recursive coalgebras in Section 3 and of well-founded coalgebras in Section 4 (using a formulation based on Jacobs' next time operator [14], which we extend from Kripke polynomial set functors to arbitrary functors). We show that every coalgebra for a monomorphism preserving functor on a complete and well-powered category has a well-founded part, and provide a new proof that this is the coreflection in the category of well-founded coalgebras (Proposition 4.19), shortening our previous proof [6]. Next we provide a new proof of Taylor's General Recursion Theorem (Theorem 5.1), generalizing this to endofunctors preserving monomorphisms on a complete and well-powered category having smooth monomorphisms (see Definition 2.8). For the category of sets, this implies that "well-founded ⇒ recursive" holds for all endofunctors, strengthening Taylor's result. We then discuss the converse: is every recursive coalgebra well-founded? Here the assumption that $F$ preserves inverse images cannot be lifted, and one needs additional assumptions. In fact, we present two results: one assumes universally smooth monomorphisms and that the functor has a pre-fixed point (see Theorem 5.5). Under these assumptions we also give a new equivalent characterization of recursiveness and well-foundedness: a coalgebra is recursive if it has a coalgebra-to-algebra morphism into the initial algebra (which exists under our assumptions), see Corollary 5.6. This characterization was previously established for finitary functors on sets [3]. The other converse of the above implication is due to Taylor using the concept of a subobject classifier (Theorem 5.8). It implies that 'recursive' and 'well-founded' are equivalent concepts for all set functors preserving inverse images. We also prove that a similar result holds for the category of vector spaces over a fixed field (Theorem 5.12).

Finally, we show in Section 6 that well-founded coalgebras are closed under coproducts, quotients and, assuming mild assumptions, under subcoalgebras.

### 2 Preliminaries

We start by recalling some background material. Except for the definitions of *algebra* and *coalgebra* in Subsection 2.1, the subsections below may be read as needed. We assume that readers are familiar with notions of basic category theory; see e.g. [2] for everything which we do not detail. We indicate monomorphisms by writing $\rightarrowtail$ and strong epimorphisms by $\twoheadrightarrow$.

**2.1 Algebras and Coalgebras.** We are concerned throughout this paper with *algebras* and *coalgebras* for an endofunctor. This means that we have an underlying category, usually written $\mathscr{A}$; frequently it is the category of sets or of vector spaces over a fixed field, and that a functor $F\colon \mathscr{A} \to \mathscr{A}$ is given. An $F$-*algebra* is a pair $(A, \alpha)$, where $\alpha\colon FA \to A$. An $F$-*coalgebra* is a pair $(A, \alpha)$, where $\alpha\colon A \to FA$. We usually drop the functor $F$. Given two algebras $(A, \alpha)$ and $(B, \beta)$, an *algebra homomorphism* from the first to the second is $h\colon A \to B$ in $\mathscr{A}$ such that $h \cdot \alpha = \beta \cdot Fh$. Similarly, a *coalgebra homomorphism* satisfies $\beta \cdot h = Fh \cdot \alpha$. We denote by $\mathsf{Coalg}\, F$ the category of all coalgebras for $F$.

**Example 2.1.** (1) The power set functor $\mathscr{P}\colon \mathsf{Set} \to \mathsf{Set}$ takes a set $X$ to the set $\mathscr{P}X$ of all subsets of it; for a morphism $f\colon X \to Y$, $\mathscr{P}f\colon \mathscr{P}X \to \mathscr{P}Y$ takes a subset $S \subseteq X$ to its direct image $f[S]$. Coalgebras $\alpha\colon X \to \mathscr{P}X$ may be identified with directed graphs on the set $X$ of vertices, and the coalgebra structure $\alpha$ describes the edges: $b \in \alpha(a)$ means that there is an edge $a \to b$ in the graph.

(2) Let $\Sigma$ be a signature, i.e. a set of operation symbols, each with a finite arity. The *polynomial functor* $H_\Sigma$ associated to $\Sigma$ assigns to a set $X$ the set

$$H_\Sigma X = \coprod_{n\in\mathbb{N}} \Sigma_n \times X^n,$$

where $\Sigma_n$ is the set of operation symbols of arity $n$. This may be identified with the set of all terms $\sigma(x_1,\dots,x_n)$, for $\sigma \in \Sigma_n$, and $x_1,\dots,x_n \in X$. Algebras for $H_\Sigma$ are the usual $\Sigma$-algebras.

(3) Deterministic automata over an input alphabet $\Sigma$ are coalgebras for the functor $FX = \{0,1\} \times X^\Sigma$. Indeed, given a set $S$ of states, a next-state map $S \times \Sigma \to S$ may be curried to $\delta\colon S \to S^\Sigma$. The set of final states yields the acceptance predicate $a\colon S \to \{0,1\}$. So an automaton may be regarded as a coalgebra $\langle a,\delta\rangle\colon S \to \{0,1\} \times S^\Sigma$.

(4) Labelled transitions systems are coalgebras for $FX = \mathscr{P}(\Sigma \times X)$.

(5) To describe linear weighted automata, i.e. weighted automata over the input alphabet $\Sigma$ with weights in a field $K$, as coalgebras, one works with the category $\mathsf{Vec}_K$ of vector spaces over $K$. A linear weighted automaton is then a coalgebra for $FX = K \times X^\Sigma$.

**2.2 Preservation Properties.** Recall that an intersection of two subobjects $s_i\colon S_i \rightarrowtail A$ $(i = 1,2)$ of a given object $A$ is given by their pullback. Analogously, (general) intersections are given by wide pullbacks. Furthermore, the inverse image of a subobject $s\colon S \rightarrowtail B$ under a morphism $f\colon A \to B$ is the subobject $t\colon T \rightarrowtail A$ obtained by a pullback of $s$ along $f$.

All of the 'usual' set functors preserve intersections and inverse images:

**Example 2.2.** (1) Every polynomial functor preserves intersections and inverse images.

(2) The power-set functor $\mathscr{P}$ preserves intersections and inverse images.

(3) Intersection-preserving set functors are closed under taking coproducts, products and composition. Similarly, for inverse images.

(4) Consider next the set functor $R$ defined by $RX = \{(x,y) \in X \times X : x \neq y\} + \{d\}$ for sets $X$. For a function $f\colon X \to Y$ put $Rf(x,y) = (f(x),f(y))$ if $f(x) \neq f(y)$, and $d$ otherwise. $R$ preserves intersections but not inverse images.

**Proposition 2.3** [27]**.** *For every set functor $F$ there exists an essentially unique set functor $\bar{F}$ which coincides with $F$ on nonempty sets and functions and preserves finite intersections (whence monomorphisms).*

**Remark 2.4.** (1) In fact, Trnková gave a construction of $\bar{F}$: she defined $\bar{F}\emptyset$ as the set of all natural transformations $C_{01} \to F$, where $C_{01}$ is the set functor with $C_{01}\emptyset = \emptyset$ and $C_{01}X = 1$ for all nonempty sets $X$. For the empty map $e\colon \emptyset \to X$ with $X \neq \emptyset$, $\bar{F}e$ maps a natural transformation $\tau\colon C_{01} \to F$ to the element given by $\tau_X\colon 1 \to FX$.

(2) The above functor $\bar{F}$ is called the *Trnková hull* of $F$. It allows us to achieve preservation of intersections for all *finitary* set functors. Intuitively, a functor on sets is finitary if its behavior is completely determined by its action on *finite* sets and functions. For a general functor, this intuition is captured by requiring that the functor preserves filtered colimits [8]. For a set functor $F$ this is equivalent to being *finitely bounded*, which is the following condition: for each element $x \in FX$ there exists a finite subset $M \subseteq X$ such that $x \in Fi[FM]$, where $i\colon M \hookrightarrow X$ is the inclusion map [7, Rem. 3.14].

**Proposition 2.5 [4, p. 66].** *The Trnková hull of a finitary set functor preserves all intersections.*

**2.3 Factorizations.** Recall that an epimorphism $e\colon A \to B$ is called *strong* if it satisfies the following *diagonal fill-in property*: given a monomorphism $m\colon C \rightarrowtail D$ and morphisms $f\colon A \to C$ and $g\colon B \to D$ such that $m \cdot f = g \cdot e$ then there exists a unique $d\colon B \to C$ such that $f = d \cdot e$ and $g = m \cdot d$.

Every complete and well-powered category has factorizations of morphisms: every morphism $f$ may be written as $f = m \cdot e$, where $e$ is a strong epimorphism and $m$ is a monomorphism [9, Prop. 4.4.3]. We call the subobject $m$ the *image* of $f$. It follows from a result in Kurz' thesis [16, Prop. 1.3.6] that factorizations of morphisms lift to coalgebras:

**Proposition 2.6 ($\mathsf{Coalg}\, F$ inherits factorizations from $\mathscr{A}$).** *Suppose that $F$ preserves monomorphisms. Then the category $\mathsf{Coalg}\, F$ has factorizations of homomorphisms $f$ as $f = m \cdot e$, where $e$ is carried by a strong epimorphism and $m$ by a monomorphism in $\mathscr{A}$. The diagonal fill-in property holds in $\mathsf{Coalg}\, F$.*

**Remark 2.7.** By a *subcoalgebra* of a coalgebra $(A, \alpha)$ we mean a subobject in $\mathsf{Coalg}\, F$ represented by a homomorphism $m\colon (B, \beta) \rightarrowtail (A, \alpha)$, where $m$ is monic in $\mathscr{A}$. Similarly, by a *strong quotient* of a coalgebra $(A, \alpha)$ we mean one represented by a homomorphism $e\colon (A, \alpha) \twoheadrightarrow (C, \gamma)$ with $e$ strongly epic in $\mathscr{A}$.

**2.4 Chains.** By a *transfinite chain* in a category $\mathscr{A}$ we understand a functor from the ordered class $\mathsf{Ord}$ of all ordinals into $\mathscr{A}$. Moreover, for an ordinal $\lambda$, a $\lambda$-*chain* in $\mathscr{A}$ is a functor from $\lambda$ to $\mathscr{A}$. A category *has colimits of chains* if for every ordinal $\lambda$ it has a colimit of every $\lambda$-chain. This includes the initial object $0$ (the case $\lambda = 0$).

**Definition 2.8.** (1) A category $\mathscr{A}$ has *smooth monomorphisms* if for every $\lambda$-chain $C$ of monomorphisms a colimit exists, its colimit cocone is formed by monomorphisms, and for every cone of $C$ formed by monomorphisms, the factorizing morphism from $\mathsf{colim}\, C$ is monic. In particuar, every morphism from $0$ is monic.

(2) $\mathscr{A}$ has *universally smooth monomorphisms* if $\mathscr{A}$ also has pullbacks, and for every morphism $f\colon X \to \mathsf{colim}\, C$, the functor $\mathscr{A}/\,\mathsf{colim}\, C \to \mathscr{A}/X$ forming pullbacks along $f$ preserves the colimit of $C$. This implies that initial object $0$ is *strict*, i.e. every morphism $f\colon X \to 0$ is an isomorphism. Indeed, consider the empty chain ($\lambda = 0$).

**Example 2.9.** (1) $\mathsf{Set}$ has universally smooth monomorphisms.

(2) $\mathsf{Vec}_K$ has smooth monomorphisms, but not universally so because the initial object is not strict.

(3) Categories in which colimits of chains and pullbacks are formed "set-like" have universally smooth monomorphisms. These include the categories of posets, graphs, topological spaces, presheaf categories, and many varieties, such as monoids, groups, and unary algebras.

(4) Every locally finitely presentable category $\mathscr{A}$ with a strict initial object (see Remark 2.12(1)) has smooth monomorphisms. This follows from [8, Prop. 1.62]. Moreover, since pullbacks commute with colimits of chains, it is easy to prove that colimits of chains are universal using the strictness of 0.

(5) The category $\mathsf{CPO}$ of complete partial orders does not have smooth monomorphisms. Indeed, consider the $\omega$-chain of linearly ordered sets $A_n = \{0, \ldots, n\} + \{\top\}$ ($\top$ a top element) with inclusion maps $A_n \to A_{n+1}$. Its colimit is the linearly ordered set $\mathbb{N} + \{\top, \top'\}$ of natural numbers with two added top elements $\top' < \top$. For the sub-cpo $\mathbb{N} + \{\top\}$, the inclusions of $A_n$ are monic and form a cocone. But the unique factorizing morphism from the colimit is not monic.

**Notation 2.10.** For every object $A$ we denote by $\mathsf{Sub}(A)$ the poset of all subobjects of $A$ (represented by monomorphisms $s\colon S \rightarrowtail A$), where $s \le s'$ if there exists $i$ with $s = s' \cdot i$. If $\mathscr{A}$ has pullbacks we have, for every morphism $f\colon A \to B$, the *inverse image operator*, viz. the monotone map $\overleftarrow{f}\colon \mathsf{Sub}(B) \to \mathsf{Sub}(A)$ assigning to a subobject $s\colon S \rightarrowtail A$ the subobject of $B$ obtained by forming the inverse image of $s$ under $f$, i.e. the pullback of $s$ along $f$.

**Lemma 2.11.** *If $\mathscr{A}$ is complete and well-powered, then $\overleftarrow{f}$ has a left adjoint given by the* (direct) image operator $\overrightarrow{f}\colon \mathsf{Sub}(A) \to \mathsf{Sub}(B)$*. It maps a subobject $t\colon T \rightarrowtail B$ to the subobject of $A$ given by the image of $f \cdot t$; in symbols we have $\overrightarrow{f}(t) \le s$ iff $t \le \overleftarrow{f}(s)$.*

**Remark 2.12.** If $\mathscr{A}$ is a complete and well-powered category, then $\mathsf{Sub}(A)$ is a complete lattice. Now suppose that $\mathscr{A}$ has smooth monomorphisms.

(1) In this setting, the unique morphism $\bot_A\colon 0 \to A$ is a monomorphism and therefore is the bottom element of the poset $\mathsf{Sub}(A)$.

(2) Furthermore, a join of a chain in $\mathsf{Sub}(A)$ is obtained by forming a colimit, in the obvious way.

(3) If $\mathscr{A}$ has universally smooth monomorphisms, then for every morphism $f\colon A \to B$, the operator $\overleftarrow{f}\colon \mathsf{Sub}(B) \to \mathsf{Sub}(A)$ preserves unions of chains.

**Remark 2.13.** Recall [1] that every endofunctor $F$ yields the *initial-algebra chain*, viz. a transfinite chain formed by the objects $F^i 0$ of $\mathscr{A}$, as follows: $F^0 0 = 0$, the initial object; $F^{i+1} 0 = F(F^i 0)$, and for a limit ordinal $i$ we take the colimit of the chain $(F^j 0)_{j<i}$. The connecting morphisms $w_{i,j}\colon F^i 0 \to F^j 0$ are defined by a similar transfinite recursion.

## 3 Recursive Coalgebras

**Assumption 3.1.** We work with a standard set theory (e.g. Zermelo-Fraenkel), assuming the Axiom of Choice. In particular, we use transfinite induction on several occasions. (We are not concerned with constructive foundations in this paper.)

Throughout this paper we assume that $\mathscr{A}$ is a complete and well-powered category $\mathscr{A}$ and that $F\colon \mathscr{A} \to \mathscr{A}$ preserves monomorphisms.

For $\mathscr{A} = \mathsf{Set}$ the condition that $F$ preserves monomorphisms may be dropped. In fact, preservation of non-empty monomorphism is sufficient in general (for a suitable notion of non-empty monomorphism) [21, Lemma 2.5], and this holds for every set functor.

The following definition of recursive coalgebras was first given by Osius [22]. Taylor [24] speaks of *coalgebras obeying the recursion scheme*. Capretta et al. [10] extended the concept to *parametrically recursive* coalgebra by dualizing completely iterative algebras [20].

**Definition 3.2.** A coalgebra $\alpha\colon A \to FA$ is called *recursive* if for every algebra $e\colon FX \to X$ there exists a unique coalgebra-to-algebra morphism $e^\dagger\colon A \to X$, i.e. a unique morphism such that the square on the left below commutes:

$$\begin{array}{ccc} A & \xrightarrow{e^\dagger} & X \\ {\scriptstyle\alpha}\downarrow & & \uparrow{\scriptstyle e} \\ FA & \xrightarrow{Fe^\dagger} & FX \end{array} \qquad\qquad \begin{array}{ccc} A & \xrightarrow{e^\dagger} & X \\ {\scriptstyle\langle\alpha,A\rangle}\downarrow & & \uparrow{\scriptstyle e} \\ FA\times A & \xrightarrow{Fe^\dagger\times A} & FX\times A \end{array}$$

$(A, \alpha)$ is called *parametrically recursive* if for every morphism $e\colon FX \times A \to X$ there is a unique morphism $e^\dagger\colon A \to X$ such that the square on the right above commutes.

**Example 3.3.** (1) A graph regarded as a coalgebra for $\mathscr{P}$ is recursive iff it has no infinite path. This is an immediate consequence of the General Recursion Theorem (see Corollary 5.6 and Example 4.5(2)).

(2) Let $\iota\colon F(\mu F) \to \mu F$ be an initial algebra. By Lambek's Lemma, $\iota$ is an isomorphism. So we have a coalgebra $\iota^{-1}\colon \mu F \to F(\mu F)$. This algebra is (parametrically) recursive. By [20, Thm. 2.8], in dual form, this is precisely the same as the terminal parametrically recursive coalgebra (see also [10, Prop. 7]).

(3) The initial coalgebra $0 \to F0$ is recursive.

(4) If $(C, \gamma)$ is recursive so is $(FC, F\gamma)$, see [10, Prop. 6].

(5) Colimits of recursive coalgebras in $\mathsf{Coalg}\, F$ are recursive. This is easy to prove, using that colimits of coalgebras are formed on the level of the underlying category.

(6) It follows from items (3)–(5) that in the initial-algebra chain from Remark 2.13 all coalgebras $w_{i,i+1}\colon F^i 0 \to F^{i+1}0$, $i \in \mathsf{Ord}$, are recursive.

(7) Every parametrically recursive coalgebra is recursive. (To see this, form for a given $e\colon FX \to X$ the morphism $e' = e \cdot \pi$, where $\pi\colon FX \times A \to FX$ is the projection.) In Corollaries 5.6 and 5.9 we will see that the converse often holds.

Here is an example where the converse fails [3]. Let $R\colon \mathsf{Set} \to \mathsf{Set}$ be the functor defined in Example 2.2(4). Also, let $C = \{0, 1\}$, and define $\gamma\colon C \to RC$ by $\gamma(0) = \gamma(1) = (0, 1)$. Then $(C, \gamma)$ is a recursive coalgebra. Indeed, for every algebra $\alpha\colon RA \to A$ the constant map $h\colon C \to A$ with $h(0) = h(1) = \alpha(d)$ is the unique coalgebra-to-algebra morphism.

However, $(C, \gamma)$ is not parametrically recursive. To see this, consider any morphism $e\colon RX \times \{0, 1\} \to X$ such that $RX$ contains more than one pair $(x_0, x_1)$, $x_0 \neq x_1$ with $e((x_0, x_1), i) = x_i$ for $i = 0, 1$. Then each such pair yields $h\colon C \to X$ with $h(i) = x_i$ making the appropriate square commutative. Thus, $(C, \gamma)$ is not parametrically recursive.

(8) Capretta et al. [11] showed that recursivity semantically models divide-and-conquer programs, as demonstrated by the example of Quicksort. For every linearly ordered set $A$ (of data elements), Quicksort is usually defined as the recursive function $q\colon A^* \to A^*$ given by

$$q(\varepsilon) = \varepsilon \qquad \text{and} \qquad q(aw) = q(w_{\leq a}) \star (aq(w_{>a})),$$

where $A^*$ is the set of all lists on $A$, $\varepsilon$ is the empty list, $\star$ is the concatenation of lists and $w_{\leq a}$ denotes the list of those elements of $w$ which are less than or equal than $a$; analogously for $w_{>a}$.

Now consider the functor $FX = 1 + A \times X \times X$ on $\mathsf{Set}$, where $1 = \{\bullet\}$, and form the coalgebra $s\colon A^* \to 1 + A \times A^* \times A^*$ given by

$$s(\varepsilon) = \bullet \qquad \text{and} \qquad s(aw) = (a, w_{\leq a}, w_{>a}) \qquad \text{for } a \in A \text{ and } w \in A^*.$$

We shall see that this coalgebra is recursive in Example 5.3. Thus, for the $F$-algebra $m : 1 + A \times A^* \times A^* \to A^*$ given by

$$m(\bullet) = \varepsilon \qquad \text{and} \qquad m(a, w, v) = w \star (av)$$

there exists a unique function $q$ on $A^*$ such that $q = m \cdot Fq \cdot s$. Notice that the last equation reflects the idea that Quicksort is a divide-and-conquer algorithm. The coalgebra structure $s$ divides a list into two parts $w_{\leq a}$ and $w_{>a}$. Then $Fq$ sorts these two smaller lists, and finally in the combine- (or conquer-) step, the algebra structure $m$ merges the two sorted parts to obtain the desired whole sorted list.

Jeannin et al. [15, Sec. 4] provide a number of recursive functions arising in programming that are determined by recursivity of a coalgebra, e.g. the gcd of integers, the Ackermann function, and the Towers of Hanoi.

### 4 The Next Time Operator and Well-Founded Coalgebras

As we have mentioned in the Introduction, the main issue of this paper is the relationship between two concepts pertaining to coalgebras: recursiveness and well-foundedness. The concept of well-foundedness is well-known for directed graphs $(G, \to)$: it means that there are no infinite directed paths $g_0 \to g_1 \to \cdots$. For a set $X$ with a relation $R$, well-foundedness means that there are no *backwards* sequences $\cdots R\, x_2\, R\, x_1\, R\, x_0$, i.e. the converse of the relation is well-founded as a graph. Taylor [24, Def. 6.2.3] gave a more general category theoretic formulation of well-foundedness. We observe here that his definition can be presented in a compact way, by using an operator that generalizes the way one thinks of the semantics of the 'next time' operator of temporal logics for non-deterministic (or even probabilistic) automata and transitions systems. It is also strongly related to the algebraic semantics of modal logic, where one passes from a graph $G$ to a function on $\mathscr{P}G$. Jacobs [14] defined and studied the 'next time' operator on coalgebras for Kripke polynomial set functors. This can be generalized to arbitrary functors as follows.

Recall that $\mathsf{Sub}(A)$ denotes the complete lattice of subobjects of $A$.

**Definition 4.1 [4, Def. 8.9].** Every coalgebra $\alpha\colon A \to FA$ induces an endofunction on $\mathsf{Sub}(A)$, called the *next time operator*

$$\bigcirc\colon\ \mathsf{Sub}(A) \to \mathsf{Sub}(A), \qquad \bigcirc(s) = \overleftarrow{\alpha}(Fs) \quad \text{for } s \in \mathsf{Sub}(A).$$

In more detail: we define $\bigcirc s$ and $\alpha(s)$ by the pullback in (4.1). (Being a pullback is indicated by the "corner" symbol.) In words, $\bigcirc$ assigns to each subobject $s\colon S \rightarrowtail A$ the inverse image of $Fs$ under $\alpha$. Since $Fs$ is a monomorphism, $\bigcirc s$ is a monomorphism and $\alpha(s)$ is (for every representation $\bigcirc s$ of that subobject of $A$) uniquely determined.

$$\begin{array}{ccc} \bigcirc S & \xrightarrow{\alpha(s)} & FS \\ {\scriptstyle \bigcirc s}\downarrow \lrcorner & & \downarrow{\scriptstyle Fs} \\ A & \xrightarrow{\alpha} & FA \end{array} \tag{4.1}$$

**Example 4.2.** (1) Let $A$ be a graph, considered as a coalgebra for $\mathscr{P}\colon \mathsf{Set} \to \mathsf{Set}$. If $S \subseteq A$ is a set of vertices, then $\bigcirc S$ is the set of vertices all of whose successors belong to $S$.

(2) For the set functor $FX = \mathscr{P}(\Sigma \times X)$ expressing labelled transition systems the operator $\bigcirc$ for a coalgebra $\alpha\colon A \to \mathscr{P}(\Sigma \times A)$ is the semantic counterpart of the next time operator of classical linear temporal logic, see e.g. Manna and Pnüeli [18]. In fact, for a subset $S \hookrightarrow A$ we have that $\bigcirc S$ consists of those states all of whose next states lie in $S$, in symbols:

$$\bigcirc S = \big\{ x \in A \mid (s, y) \in \alpha(x) \text{ implies } y \in S, \text{ for all } s \in \Sigma \big\}.$$

The next time operator allows a compact definition of well-foundedness as characterized by Taylor [24, Exercise VI.17] (see also [6, Corollary 2.19]):

**Definition 4.3.** A coalgebra is *well-founded* if $id_A$ is the only fixed point of its next time operator.

**Remark 4.4.** (1) Let us call a subcoalgebra $m\colon (B, \beta) \rightarrowtail (A, \alpha)$ *cartesian* provided that the square (4.2) is a pullback. Then $(A, \alpha)$ is well-founded iff it has no proper cartesian subcoalgebra. That is, if $m\colon (B, \beta) \rightarrowtail (A, \alpha)$ is a cartesian subcoalgebra, then $m$ is an isomorphism. Indeed, the fixed points of next time are precisely the

$$\begin{array}{ccc} B & \xrightarrow{\beta} & FB \\ {\scriptstyle m}\downarrow \lrcorner & & \downarrow{\scriptstyle Fm} \\ A & \xrightarrow{\alpha} & FA \end{array} \tag{4.2}$$

cartesian subcoalgebras.

(2) A coalgebra is well-founded iff $\bigcirc$ has a unique pre-fixed point $\bigcirc m \le m$. Indeed, since $\mathsf{Sub}(A)$ is a complete lattice, the least fixed point of a monotone map is its least pre-fixed point. Taylor's definition [24, Def. 6.3.2] uses that property: he calls a coalgebra well-founded iff $\bigcirc$ has no proper subobject as a pre-fixed point.

**Example 4.5.** (1) Consider a graph as a coalgebra $\alpha\colon A \to \mathscr{P}A$ for the power-set functor (see Example 2.1). A subcoalgebra is a subset $m\colon B \hookrightarrow A$ such that with every vertex $v$ it contains all neighbors of $v$. The coalgebra structure $\beta\colon B \to \mathscr{P}B$ is then the domain-codomain restriction of $\alpha$. To say that $B$ is a cartesian subcoalgebra means that whenever a vertex of $A$ has all neighbors in $B$, it also lies in $B$. It follows that $(A,\alpha)$ is well-founded iff it has no infinite directed path, see [24, Example 6.3.3].

(2) If $\mu F$ exists, then as a coalgebra it is well-founded. Indeed, in every pullback (4.2), since $\iota^{-1}$ (as $\alpha$) is invertible, so is $\beta$. The unique algebra homomorphism from $\mu F$ to the algebra $\beta^{-1}\colon FB \to B$ is clearly inverse to $m$.

(3) If a set functor $F$ fulfils $F\emptyset = \emptyset$, then the only well-founded coalgebra is the empty one. Indeed, this follows from the fact that the empty coalgebra is a fixed point of $\bigcirc$. For example, a deterministic automaton over the input alphabet $\Sigma$, as a coalgebra for $FX = \{0,1\} \times X^{\Sigma}$, is well-founded iff it is empty.

(4) A non-deterministic automaton may be considered as a coalgebra for the set functor $FX = \{0,1\} \times (\mathscr{P}X)^{\Sigma}$. It is well-founded iff the state transition graph is well-founded (i.e. has no infinite path). This follows from Corollary 4.10 below.

(5) A linear weighted automaton, i.e. a coalgebra for $FX = K \times X^{\Sigma}$ on $\mathsf{Vec}_K$, is well-founded iff every path in its state transition graph eventually leads to 0. This means that every path starting in a given state leads to the state 0 after finitely many steps (where it stays).

**Notation 4.6.** Given a set functor $F$, we define for every set $X$ the map $\tau_X\colon FX \to \mathscr{P}X$ assigning to every element $x \in FX$ the intersection of all subsets $m\colon M \hookrightarrow X$ such that $x$ lies in the image of $Fm$:

$$\tau_X(x) = \bigcap \{m \mid m\colon M \hookrightarrow X \text{ satisfies } x \in Fm[FM]\}. \tag{4.3}$$

Recall that a functor *preserves intersections* if it preserves (wide) pullbacks of families of monomorphisms.

Gumm [13, Thm. 7.3] observed that for a set functor preserving intersections, the maps $\tau_X\colon FX \to \mathscr{P}X$ in (4.3) form a "subnatural" transformation from $F$ to the power-set functor $\mathscr{P}$. Subnaturality means that (although these maps do not form a natural transformation in general) for every monomorphism $i\colon X \to Y$ we have a commutative square:

$$\begin{array}{ccc} FX & \xrightarrow{\tau_X} & \mathscr{P}X \\ {\scriptstyle Fi}\downarrow & & \downarrow{\scriptstyle \mathscr{P}i} \\ FY & \xrightarrow{\tau_Y} & \mathscr{P}Y \end{array} \tag{4.4}$$

**Remark 4.7.** As shown in [13, Thm. 7.4] and [23, Prop. 7.5], a set functor $F$ preserves intersections iff the squares in (4.4) above are pullbacks. Moreover, *loc. cit.* and [13, Thm. 8.1] prove that $\tau\colon F \to \mathscr{P}$ is a natural transformation, provided $F$ preserves inverse images and intersections.

**Definition 4.8.** Let $F$ be a set functor. For every coalgebra $\alpha\colon A \to FA$ its *canonical graph* is the following coalgebra for $\mathscr{P}$: $A \xrightarrow{\alpha} FA \xrightarrow{\tau_A} \mathscr{P}A$.

Thanks to the subnaturality of $\tau$ one obtains the following results.

**Proposition 4.9.** *For every set functor $F$ preserving intersections, the next time operator of a coalgebra $(A, \alpha)$ coincides with that of its canonical graph.*

**Corollary 4.10 [24, Rem. 6.3.4].** *A coalgebra for a set functor preserving intersections is well-founded iff its canonical graph is well-founded.*

**Example 4.11.** (1) For a (deterministic or non-deterministic) automaton, the canonical graph has an edge from $s$ to $t$ iff there is a transition from $s$ to $t$ for some input letter. Thus, we obtain the characterization of well-foundedness as stated in Example 4.5(3) and (4).

(2) Every polynomial functor $H_\Sigma\colon \mathsf{Set} \to \mathsf{Set}$ preserves intersections. Thus, a coalgebra $(A, \alpha)$ is well-founded if there are no infinite paths in its canonical graph. The canonical graph of $A$ has an edge from $a$ to $b$ if $\alpha(a)$ is of the form $\sigma(c_1, \dots, c_n)$ for some $\sigma \in \Sigma_n$ and if $b$ is one of the $c_i$'s.

(3) Thus, for the functor $FX = 1 + A \times X \times X$, the coalgebra $(A^*, s)$ of Example 3.3(8) is easily seen to be well-founded via its canonical graph. Indeed, this graph has for every list $w$ one outgoing edge to the list $w_{\leq a}$ and one to $w_{>a}$ for every $a \in A$. Hence, this is a well-founded graph.

**Lemma 4.12.** *The next time operator is monotone: if $m \leq n$, then $\bigcirc m \leq \bigcirc n$.*

**Lemma 4.13.** *Let $\alpha\colon A \to FA$ be a coalgebra and $m\colon B \rightarrowtail A$ a subobject.*

(1) *There is a coalgebra structure $\beta\colon B \to FB$ for which $m$ gives a subcoalgebra of $(A, \alpha)$ iff $m \leq \bigcirc m$.*

(2) *There is a coalgebra structure $\beta\colon B \to FB$ for which $m$ gives a cartesian subcoalgebra of $(A, \alpha)$ iff $m = \bigcirc m$.*

**Lemma 4.14.** *For every coalgebra homomorphism $f\colon (B, \beta) \to (A, \alpha)$ we have*

$$\bigcirc_\beta \cdot \overleftarrow{f} \leq \overleftarrow{f} \cdot \bigcirc_\alpha,$$

*where $\bigcirc_\alpha$ and $\bigcirc_\beta$ denote the next time operators of the coalgebras $(A, \alpha)$ and $(B, \beta)$, respectively, and $\leq$ is the pointwise order.*

**Corollary 4.15.** *For every coalgebra homomorphism $f\colon (B, \beta) \to (A, \alpha)$ we have $\bigcirc_\beta \cdot \overleftarrow{f} = \overleftarrow{f} \cdot \bigcirc_\alpha$, provided that either*

(1) *$f$ is a monomorphism in $\mathscr{A}$ and $F$ preserves finite intersections, or*
(2) *$F$ preserves inverse images.*

**Definition 4.16 [4].** The *well-founded part* of a coalgebra is its largest well-founded subcoalgebra.

The well-founded part of a coalgebra always exists and is the coreflection in the category of well-founded coalgebras [6, Prop. 2.27]. We provide a new, shorter proof of this fact. The well-founded part is obtained by the following:

**Construction 4.17 [6, Not. 2.22].** Let $\alpha\colon A \to FA$ be a coalgebra. We know that $\mathsf{Sub}(A)$ is a complete lattice and that the next time operator $\bigcirc$ is monotone (see Lemma 4.12). Hence, by the Knaster-Tarski fixed point theorem, $\bigcirc$ has a least fixed point, which we denote by $a^*\colon A^* \rightarrowtail A$.

By Lemma 4.13(2), we know that there is a coalgebra structure $\alpha^*\colon A^* \to FA^*$ so that $a^*\colon (A^*, \alpha^*) \rightarrowtail (A, \alpha)$ is the smallest cartesian subcoalgebra of $(A, \alpha)$.

**Proposition 4.18.** *For every coalgebra $(A, \alpha)$, the coalgebra $(A^*, \alpha^*)$ is well-founded.*

*Proof.* Let $m\colon (B, \beta) \rightarrowtail (A^*, \alpha^*)$ be a cartesian subcoalgebra. By Lemma 4.13, $a^* \cdot m\colon B \to A$ is a fixed point of $\bigcirc$. Since $a^*$ is the least fixed point, we have $a^* \leq a^* \cdot m$, i.e. $a^* = a^* \cdot m \cdot x$ for some $x\colon A^* \rightarrowtail B$. Since $a^*$ is monic, we thus have $m \cdot x = id_{A^*}$. So $m$ is a monomorphism and a split epimorphism, whence an isomorphism. $\square$

**Proposition 4.19.** *The full subcategory of $\mathsf{Coalg}\, F$ given by well-founded coalgebras is coreflective. In fact, the well-founded coreflection of a coalgebra $(A, \alpha)$ is its well-founded part $a^*\colon (A^*, \alpha^*) \rightarrowtail (A, \alpha)$.*

*Proof.* We are to prove that for every coalgebra homomorphism $f\colon (B, \beta) \to (A, \alpha)$, where $(B, \beta)$ is well-founded, there exists a coalgebra homomorphism $f^\sharp\colon (B, \beta) \to (A^*, \alpha^*)$ such that $a^* \cdot f^\sharp = f$. The uniqueness is easy.

For the existence of $f^\sharp$, we first observe that $\overleftarrow{f}(a^*)$ is a pre-fixed point of $\bigcirc_\beta$: indeed, using Lemma 4.14 we have $\bigcirc_\beta(\overleftarrow{f}(a^*)) \leq \overleftarrow{f}(\bigcirc_\alpha(a^*)) = \overleftarrow{f}(a^*)$. By Remark 4.4(2), we therefore have $id_B \ = b^* \leq \overleftarrow{f}(a^*)$ in $\mathsf{Sub}(B)$. Using the adjunction of Lemma 2.11, we have $\overrightarrow{f}(id_B) \leq a^*$ in $\mathsf{Sub}(A)$. Now factorize $f$ as $B \overset{e}{\twoheadrightarrow} C \overset{m}{\rightarrowtail} A$. We have $\overrightarrow{f}(id_B) = m$, and we then obtain $m = \overrightarrow{f}(id_B) \leq a^*$, i.e. there exists a morphism $h\colon C \rightarrowtail A^*$ such that $a^* \cdot h = m$. Thus, $f^\sharp = h \cdot e\colon B \to A^*$ is a morphism satisfying $a^* \cdot f^\sharp = a^* \cdot h \cdot e = m \cdot e = f$. It follows that $f^\sharp$ is a coalgebra homomorphism from $(B, \beta)$ to $(A^*, \alpha^*)$ since $f$ and $a^*$ are and $F$ preserves monomorphisms. $\square$

**Construction 4.20 [6, Not. 2.22].** Let $(A, \alpha)$ be a coalgebra. We obtain $a^*$, the least fixed point of $\bigcirc$, as the join of the following transfinite chain of subobjects $a_i\colon A_i \rightarrowtail A$, $i \in \mathsf{Ord}$. First, put $a_0 = \bot_A$, the least subobject of $A$. Given $a_i\colon A_i \rightarrowtail A$, put $a_{i+1} = \bigcirc a_i\colon A_{i+1} = \bigcirc A_i \rightarrowtail A$. For every limit ordinal $j$, put $a_j = \bigvee_{i<j} a_i$. Since $\mathsf{Sub}(A)$ is a set, there exists an ordinal $i$ such that $a_i = a^*\colon A^* \rightarrowtail A$.

**Remark 4.21.** Note that, whenever monomorphisms are smooth, we have $A_0 = 0$ and the above join $a_j$ is obtained as the colimit of the chain of the subobject $a_i \colon A_i \rightarrowtail A$, $i < j$ (see Remark 2.12).

If $F$ is a finitary functor on a locally finitely presentable category, then the least ordinal $i$ with $a^* = a_i$ is at most $\omega$, but in general one needs transfinite iteration to reach a fixed point.

**Example 4.22.** Let $(A, \alpha)$ be a graph regarded as a coalgebra for $\mathscr{P}$ (see Example 2.1). Then $A_0 = \emptyset$, $A_1$ is formed by all leaves; i.e. those nodes with no neighbors, $A_2$ by all leaves and all nodes such that every neighbor is a leaf, etc. We see that a node $x$ lies in $A_{i+1}$ iff every path starting in $x$ has length at most $i$. Hence $A^* = A_\omega$ is the set of all nodes from which no infinite paths start.

We close with a general fact on well-founded parts of *fixed points* (i.e. (co)algebras whose structure is invertible). The following result generalizes [15, Cor. 3.4], and it also appeared before for functors preserving finite intersections [4, Theorem 8.16 and Remark 8.18]. Here we lift the latter assumption (see [5, Theorem 7.6] for the new proof):

**Theorem 4.23.** *Let $\mathscr{A}$ be a complete and well-powered category with smooth monomorphisms. For $F$ preserving monomorphisms, the well-founded part of every fixed point is an initial algebra. In particular, the only well-founded fixed point is the initial algebra.*

**Example 4.24.** We illustrate that for a set functor $F$ preserving monomorphisms, the well-founded part of the terminal coalgebra is the initial algebra. Consider $FX = A \times X + 1$. The terminal coalgebra is the set $A^\infty \cup A^*$ of finite and infinite sequences from the set $A$. The initial algebra is $A^*$. It is easy to check that $A^*$ is the well-founded part of $A^\infty \cup A^*$.

### 5 The General Recursion Theorem and its Converse

The main consequence of well-foundedness is parametric recursivity. This is Taylor's General Recursion Theorem [24, Theorem 6.3.13]. Taylor assumed that $F$ preserves inverse images. We present a new proof for which it is sufficient that $F$ preserves monomorphisms, assuming those are smooth.

**Theorem 5.1 (General Recursion Theorem).** *Let $\mathscr{A}$ be a complete and wellpowered category with smooth monomorphisms. For $F \colon \mathscr{A} \to \mathscr{A}$ preserving monomorphisms, every well-founded coalgebra is parametrically recursive.*

*Proof sketch.* (1) Let $(A, \alpha)$ be well-founded. We first prove that it is recursive. We use the subobjects $a_i \colon A_i \rightarrowtail A$ of Construction 4.20[4], the corresponding

[4] One might object to this use of transfinite recursion, since Theorem 5.1 itself could be used as a justification for transfinite recursion. Let us emphasize that we are not presenting Theorem 5.1 as a foundational contribution. We are building on the classical theory of transfinite recursion.

morphisms $\alpha(a_i)\colon A_{i+1} = \bigcirc A_i \to FA_i$ (cf. Definition 4.3), and the recursive coalgebras $(F^i 0, w_{i,i+1})$ of Example 3.3(6). We obtain a natural transformation $h$ from the chain $(A_i)$ in Construction 4.20 to the initial-algebra chain $(F^i 0)$ (see Remark 2.13) by transfinite recursion.

Now for every algebra $e\colon FX \to X$, we obtain a unique coalgebra-to-algebra morphism $f_i\colon F^i 0 \to X$, i.e. we have that $f_i = e \cdot Ff_i \cdot w_{i,i+1}$. Since $(A, \alpha)$ is well-founded, we know that $\alpha = \alpha^* = \alpha(a_i)$ for some $i$. From this it is not difficult to prove that $f_i \cdot h_i$ is a coalgebra-to-algebra morphism from $(A, \alpha)$ to $(X, e)$.

In order to prove uniqueness, we prove by transfinite induction that for any given coalgebra-to-algebra homomorphism $e^\dagger$, one has $e^\dagger \cdot a_j = f_j \cdot h_j \cdot a_j$ for every ordinal number $j$. Then for the above ordinal number $i$ with $a_i = id_A$, we have $e^\dagger = f_i \cdot h_i$, as desired. This shows that $(A, \alpha)$ is recursive.

(2) We prove that $(A, \alpha)$ is parametrically recursive. Consider the coalgebra $\langle \alpha, id_A \rangle\colon A \to FA \times A$ for $F(-) \times A$. This functor preserves monomorphisms since $F$ does and monomorphisms are closed under products. The next time operator $\bigcirc$ on $\mathsf{Sub}(A)$ is the same for both coalgebras since the square (4.1) is a pullback if and only if the square on the right below is one.

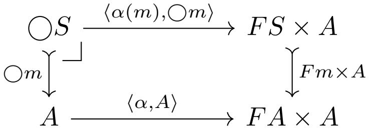

Since $id_A$ is the unique fixed point of $\bigcirc$ w.r.t. $F$ (see Definition 4.3), it is also the unique fixed point of $\bigcirc$ w.r.t. $F(-) \times A$. Thus, $(A, \langle \alpha, id_A \rangle)$ is a well-founded coalgebra for $F(-) \times A$. By the previous argument, this coalgebra is thus recursive for $F(-) \times A$; equivalently, $(A, \alpha)$ is parametrically recursive for $F$. $\square$

**Theorem 5.2.** *For every endofunctor on* $\mathsf{Set}$ *or* $\mathsf{Vec}_K$ *(vector spaces and linear maps), every well-founded coalgebra is parametrically recursive.*

*Proof sketch.* For $\mathsf{Set}$, we apply Theorem 5.1 to the Trnková hull $\bar{F}$ (see Proposition 2.3), noting that $F$ and $\bar{F}$ have the same (non-empty) coalgebras. Moreover, one can show that every well-founded (or recursive) $F$-coalgebra is a well-founded (recursive, resp.) $\bar{F}$-coalgebra. For $\mathsf{Vec}_K$, observe that monomorphisms split and are therefore preserved by every endofunctor $F$. $\square$

**Example 5.3.** We saw in Example 4.11(3) that for $FX = 1 + A \times X \times X$ the coalgebra $(A, s)$ from Example 3.3(8) is well-founded, and therefore it is (parametrically) recursive.

**Example 5.4.** Well-founded coalgebras need not be recursive when $F$ does not preserve monomorphisms. We take $\mathscr{A}$ to be the category of *sets with a predicate*, i.e. pairs $(X, A)$, where $A \subseteq X$. Morphisms $f\colon (X, A) \to (Y, B)$ satisfy $f[A] \subseteq B$. Denote by $\mathbb{1}$ the terminal object $(1, 1)$. We define an endofunctor $F$ by $F(X, \emptyset) = (X + 1, \emptyset)$, and for $A \neq \emptyset$, $F(X, A) = \mathbb{1}$. For a morphism $f\colon (X, A) \to (Y, B)$, put $Ff = f + id$ if $A = \emptyset$; if $A \neq \emptyset$, then also $B \neq \emptyset$ and $Ff$ is $id\colon \mathbb{1} \to \mathbb{1}$.

The terminal coalgebra is $id\colon \mathbb{1} \to \mathbb{1}$, and it is easy to see that it is well-founded. But it is not recursive: there are no coalgebra-to-algebra morphisms into an algebra of the form $F(X, \emptyset) \to (X, \emptyset)$.

We next prove a converse to Theorem 5.1: "recursive $\implies$ well-founded". Related results appear in Taylor [23, 24], Adámek et al. [3] and Jeannin et al. [15].

Recall universally smooth monomorphisms from Definition 2.8(2). A *pre-fixed point* of $F$ is a monic algebra $\alpha\colon FA \rightarrowtail A$.

**Theorem 5.5.** *Let $\mathscr{A}$ be a complete and wellpowered category with universally smooth monomorphisms, and suppose that $F\colon \mathscr{A} \to \mathscr{A}$ preserves inverse images and has a pre-fixed point. Then every recursive coalgebra is well-founded.*

*Proof.* (1) We first observe that an initial algebra exists. This follows from results by Trnková et al. [25] as we now briefly recall. Recall the initial-algebra chain from Remark 2.13. Let $\beta\colon FB \rightarrowtail B$ be a pre-fixed point. Then there is a unique cocone $\beta_i\colon F^i 0 \to B$ satisfying $\beta_{i+1} = \beta \cdot F\beta_i$. Moreover, each $\beta_i$ is monomorphic. Since $B$ has only a set of subobjects, there is some $\lambda$ such that for every $i > \lambda$, all of the morphisms $\beta_i$ represent the same subobject of $B$. Consequently, $w_{\lambda,\lambda+1}$ of Remark 2.13 is an isomorphism, due to $\beta_\lambda = \beta_{\lambda+1} \cdot w_{\lambda,\lambda+1}$. Then $\mu F = F^\lambda 0$ with the structure $\iota = w_{\lambda,\lambda+1}^{-1}\colon F(\mu F) \to \mu F$ is an initial algebra.
(2) Now suppose that $(A, \alpha)$ is a recursive coalgebra. Then there exists a unique coalgebra homomorphism $h\colon (A, \alpha) \to (\mu F, \iota^{-1})$. Let us abbreviate $w_{i\lambda}$ by $c_i\colon F^i 0 \rightarrowtail \mu F$, and recall the subobjects $a_i\colon A_i \rightarrowtail A$ from Construction 4.20. We will prove by transfinite induction that $a_i$ is the inverse image of $c_i$ under $h$; in symbols: $a_i = \overleftarrow{h}(c_i)$ for all ordinals $i$. Then it follows that $a_\lambda$ is an isomorphism, since so is $c_\lambda$, whence $(A, \alpha)$ is well-founded.

In the base case $i = 0$ this is clear since $A_0 = W_0 = 0$ is a strict initial object.

For the isolated step we compute the pullback of $c_{i+1}\colon W_{i+1} \to \mu F$ along $h$ using the following diagram:

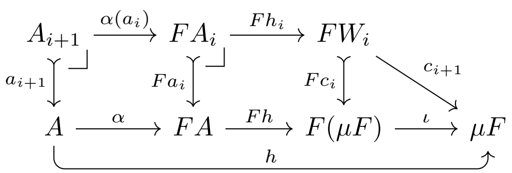

By the induction hypothesis and since $F$ preserves inverse images, the middle square above is a pullback. Since the structure map $\iota$ of the initial algebra is an isomorphism, it follows that the middle square pasted with the right-hand triangle is also a pullback. Finally, the left-hand square is a pullback by the definition of $a_{i+1}$. Thus, the outside of the above diagram is a pullback, as required.

For a limit ordinal $j$, we know that $a_j = \bigvee_{i<j} a_i$ and similarly, $c_j = \bigvee_{i<j} c_i$ since $W_j = \mathsf{colim}_{i<j} W_j$ and monomorphisms are smooth (see Remark 2.12(2)). Using Remark 2.12(3) and the induction hypothesis we thus obtain $\overleftarrow{h}(c_j) = \overleftarrow{h}\left(\bigvee_{i<j} c_i\right) = \bigvee_{i<j} \overleftarrow{h}(c_i) = \bigvee_{i<j} a_i = a_j$. $\square$

**Corollary 5.6.** *Let $\mathscr{A}$ and $F$ satisfy the assumptions of Theorem 5.5. Then the following properties of a coalgebra are equivalent:*

(1) *well-foundedness,*

(2) *parametric recursiveness,*

(3) *recursiveness,*

(4) *existence of a homomorphism into $(\mu F, \iota^{-1})$,*

(5) *existence of a homomorphism into a well-founded coalgebra.*

*Proof sketch.* We already know (1) ⇒ (2) ⇒ (3). Since $F$ has an initial algebra (as proved in Theorem 5.5), the implication (3) ⇒ (4) follows from Example 3.3(2). In Theorem 5.5 we also proved (4) ⇒ (1). The implication (4) ⇒ (5) follows from Example 4.5(2). Finally, it follows from [6, Remark 2.40] that $(\mu F, \iota^{-1})$ is a terminal well-founded coalgebra, whence (5) ⇒ (4). □

**Example 5.7.** (1) The category of many-sorted sets satisfies the assumptions of Theorem 5.5, and polynomial endofunctors on that category preserve inverse images. Thus, we obtain Jeannin et al.'s result [15, Thm. 3.3] that (1)–(4) in Corollary 5.6 are equivalent as a special instance.

(2) The implication (4) ⇒ (3) in Corollary 5.6 does not hold for vector spaces. In fact, for the identity functor on $\mathsf{Vec}_K$ we have $\mu Id = (0, id)$. Hence, every coalgebra has a homomorphism into $\mu Id$. However, not every coalgebra is recursive, e.g. the coalgebra $(K, id)$ admits many coalgebra-to-algebra morphisms to the algebra $(K, id)$. Similarly, the implication (4) ⇒ (1) does not hold.

We also wish to mention a result due to Taylor [23, Rem. 3.8]. It uses the concept of a *subobject classifier* originating in [17] and prominent in topos theory. This is an object $\Omega$ with a subobject $t\colon 1 \rightarrowtail \Omega$ such that for every subobject $b\colon B \rightarrowtail A$ there is a unique $\hat{b}\colon A \to \Omega$ such that $b$ is the inverse image of $t$ under $\hat{b}$. By definition, every elementary topos has a subobject classifier, in particular every category $\mathsf{Set}^{\mathscr{C}}$ with $\mathscr{C}$ small.

Our standing assumption that $\mathscr{A}$ is a complete and well-powered category is not needed for the next result: finite limits are sufficient.

**Theorem 5.8 (Taylor [23]).** *Let $F$ be an endofunctor preserving inverse images on a finitely complete category with a subobject classifier. Then every recursive coalgebra is well-founded.*

**Corollary 5.9.** *For every set functor preserving inverse images, the following properties of a coalgebra are equivalent:*

$$\textit{well-foundedness} \iff \textit{parametric recursiveness} \iff \textit{recursiveness.}$$

**Example 5.10.** The hypothesis in Theorems 5.5 and 5.8 that the functor preserves inverse images cannot be lifted. In order to see this, we consider the functor $R\colon \mathsf{Set} \to \mathsf{Set}$ of Example 2.2(4). It preserves monomorphisms but not inverse images. The coalgebra $A = \{0, 1\}$ with the structure $\alpha$ constant to $(0, 1)$ is recursive: given an algebra $\beta\colon RB \to B$, the unique coalgebra-to-algebra homomorphism $h\colon \{0,1\} \to B$ is given by $h(0) = h(1) = \beta(d)$. But $A$ is not well-founded: $\emptyset$ is a cartesian subcoalgebra.

Recall that an initial algebra $(\mu F, \iota)$ is also considered as a coalgebra $(\mu F, \iota^{-1})$. Taylor [23, Cor. 9.9] showed that, for functors preserving inverse images, the terminal well-founded coalgebra is the initial algebra. Surprisingly, this result is true for *all* set functors.

**Theorem 5.11 [6, Thm. 2.46].** *For every set functor, a terminal well-founded coalgebra is precisely an initial algebra.*

**Theorem 5.12.** *For every functor on* $\mathsf{Vec}_K$ *preserving inverse images, the following properties of a coalgebra are equivalent:*

*well-foundedness* $\iff$ *parametric recursiveness* $\iff$ *recursiveness.*

### 6 Closure Properties of Well-founded Coalgebras

In this section we will see that strong quotients and subcoalgebras (see Remark 2.7) of well-founded coalgebras are well-founded again. We mention the following corollary to Proposition 4.19. For endofunctors on sets preserving inverse images this was stated by Taylor [24, Exercise VI.16]:

**Proposition 6.1.** *The subcategory of* $\mathsf{Coalg}\,F$ *formed by all well-founded coalgebras is closed under strong quotients and coproducts in* $\mathsf{Coalg}\,F$*.*

This follows from a general result on coreflective subcategories [2, Thm. 16.8]: the category $\mathsf{Coalg}\,F$ has the factorization system of Proposition 2.6, and its full subcategory of well-founded coalgebras is coreflective with monomorphic coreflections (see Proposition 4.19). Consequently, it is closed under strong quotients and colimits.

We prove next that, for an endofunctor preserving finite intersections, well-founded coalgebras are closed under subcoalgebras provided that the complete lattice $\mathsf{Sub}(A)$ is a *frame*. This means that for every subobject $m\colon B \rightarrowtail A$ and every family $m_i$ $(i \in I)$ of subobjects of $A$ we have $m \wedge \bigvee_{i\in I} m_i = \bigvee_{i\in I}(m \wedge m_i)$. Equivalently, $\overleftarrow{m}\colon \mathsf{Sub}(A) \to \mathsf{Sub}(B)$ (see Notation 2.10) has a right adjoint $m_*\colon \mathsf{Sub}(B) \to \mathsf{Sub}(A)$.

This property holds for $\mathsf{Set}$ as well as for the categories of posets, graphs, topological spaces, and presheaf categories $\mathsf{Set}^{\mathscr{C}}$, $\mathscr{C}$ small. Moreover, it holds for every Grothendieck topos. The categories of complete partial orders and $\mathsf{Vec}_K$ do not satisfy this requirement.

**Proposition 6.2.** *Suppose that* $F$ *preserves finite intersections, and let* $(A, \alpha)$ *be a well-founded coalgebra such that* $\mathsf{Sub}(A)$ *a frame. Then every subcoalgebra of* $(A, \alpha)$ *is well-founded.*

*Proof.* Let $m\colon (B,\beta) \rightarrowtail (A,\alpha)$ be a subcoalgebra. We will show that the only pre-fixed point of $\bigcirc_\beta$ is $id_B$ (cf. Remark 4.4(2)). Suppose $s\colon S \rightarrowtail B$ fulfils $\bigcirc_\beta(s) \leq s$. Since $F$ preserves finite intersections, we have $\overleftarrow{m} \cdot \bigcirc_\alpha = \bigcirc_\beta \cdot \overleftarrow{m}$ by Corollary 4.15(1). The counit of the above adjunction $\overleftarrow{m} \dashv m_*$ yields $\overleftarrow{m}(m_*(s)) \leq s$, so that we obtain $\overleftarrow{m}(\bigcirc_\alpha(m_*(s))) = \bigcirc_\beta(\overleftarrow{m}(m_*(s))) \leq \bigcirc_\beta(s) \leq s$. Using again the adjunction $\overleftarrow{m} \dashv m_*$, we have equivalently that $\bigcirc_\alpha(m_*(s)) \leq m_*(s)$; i.e. $m_*(s)$ is a pre-fixed point of $\bigcirc_\alpha$. Since $(A,\alpha)$ is well-founded, Corollary 4.15(1) implies that $m_*(s) = id_A$. Since $\overleftarrow{m}$ is also a right adjoint and therefore preserves the top element of $\mathsf{Sub}(B)$, we thus obtain $id_B = \overleftarrow{m}(id_A) = \overleftarrow{m}(m_*(s)) \leq s$. □

**Remark 6.3.** Given a set functor $F$ preserving inverse images, a much better result was proved by Taylor [24, Corollary 6.3.6]: for every coalgebra homomorphism $f\colon (B,\beta) \to (A,\alpha)$ with $(A,\alpha)$ well-founded so is $(B,\beta)$. In fact, our proof above is essentially Taylor's.

**Corollary 6.4.** *If a set functor preserves finite intersections, then subcoalgebras of well-founded coalgebras are well-founded.*

Trnková [26] proved that every set functor preserves all *nonempty* finite intersections. However, this does not suffice for Corollary 6.4:

**Example 6.5.** A well-founded coalgebra for a set functor can have non-well-founded subcoalgebras. Let $F\emptyset = 1$ and $FX = 1+1$ for all nonempty sets $X$, and let $Ff = \mathsf{inl}\colon 1 \to 1+1$ be the left-hand injection for all maps $f\colon \emptyset \to X$ with $X$ nonempty. The coalgebra $\mathsf{inr}\colon 1 \to F1$ is not well-founded because its empty subcoalgebra is cartesian. However, this is a subcoalgebra of $id\colon 1+1 \to 1+1$ (via the embedding $\mathsf{inr}$), and the latter is well-founded.

The fact that subcoalgebras of a well-founded coalgebra are well-founded does not necessarily need the assumption that $\mathsf{Sub}(A)$ is a frame. Instead, one may assume that the class of morphisms is universally smooth:

**Theorem 6.6.** *If $\mathscr{A}$ has universally smooth monomorphisms and $F$ preserves finite intersections, every subcoalgebra of a well-founded coalgebra is well-founded.*

### 7 Conclusions

Well-founded coalgebras introduced by Taylor [24] have a compact definition based on an extension of Jacobs' 'next time' operator. Our main contribution is a new proof of Taylor's General Recursion Theorem that every well-founded coalgebra is recursive, generalizing this result to all endofunctors preserving monomorphisms on a complete and well-powered category with smooth monomorphisms. For functors preserving inverse images, we also have seen two variants of the converse implication "recursive ⇒ well-founded", under additional hypothesis: one due to Taylor for categories with a subobject classifier, and the second one provided that the category has universally smooth monomorphisms and the functor has a pre-fixed point. Various counterexamples demonstrate that all our hypotheses are necessary.
